# Multi-Omics Profiling of Hypertrophic Cardiomyopathy Reveals Altered Mechanisms in Mitochondrial Dynamics and Excitation–Contraction Coupling

**DOI:** 10.3390/ijms24054724

**Published:** 2023-03-01

**Authors:** Jarrod Moore, Jourdan Ewoldt, Gabriela Venturini, Alexandre C. Pereira, Kallyandra Padilha, Matthew Lawton, Weiwei Lin, Raghuveera Goel, Ivan Luptak, Valentina Perissi, Christine E. Seidman, Jonathan Seidman, Michael T. Chin, Christopher Chen, Andrew Emili

**Affiliations:** 1 Center for Network Systems Biology, Department of Biochemistry, Boston University School of Medicine, Boston, MA 02118, USA; 2 Department of Biomedical Engineering, Boston University, Boston, MA 02218, USA; 3 Department of Genetics, Harvard Medical School, Boston, MA 02115, USA; 4 Laboratory of Genetics and Molecular Cardiology, Clinical Hospital, Faculty of Medicine, University of São Paulo, Sao Paulo 05508-000, Brazil; 5 Myocardial Biology Unit, Boston University School of Medicine, Boston, MA 02118, USA; 6 Division of Cardiovascular Medicine, Brigham and Women’s Hospital, Boston, MA 02115, USA; 7 Howard Hughes Medical Institute, Chevy Chase, MD 20815, USA; 8 Molecular Cardiology Research Institute, Tufts Medical Center, Boston, MA 02145, USA; 9 Wyss Institute for Biologically Inspired Engineering, Harvard University, Boston, MA 02115, USA

**Keywords:** hypertrophic cardiomyopathy, human induced pluripotent stem-cell-derived cardiomyocytes, myectomy, liquid chromatography–tandem mass spectrometry, phosphoproteomics, metabolomics, network systems biology

## Abstract

Hypertrophic cardiomyopathy is one of the most common inherited cardiomyopathies and a leading cause of sudden cardiac death in young adults. Despite profound insights into the genetics, there is imperfect correlation between mutation and clinical prognosis, suggesting complex molecular cascades driving pathogenesis. To investigate this, we performed an integrated quantitative multi-omics (proteomic, phosphoproteomic, and metabolomic) analysis to illuminate the early and direct consequences of mutations in myosin heavy chain in engineered human induced pluripotent stem-cell-derived cardiomyocytes relative to late-stage disease using patient myectomies. We captured hundreds of differential features, which map to distinct molecular mechanisms modulating mitochondrial homeostasis at the earliest stages of pathobiology, as well as stage-specific metabolic and excitation-coupling maladaptation. Collectively, this study fills in gaps from previous studies by expanding knowledge of the initial responses to mutations that protect cells against the early stress prior to contractile dysfunction and overt disease.

## 1. Introduction

Hypertrophic cardiomyopathy (HCM) is among the most common inherited cardiomyopathies and, historically, a leading cause of sudden cardiac death in young adults [1,2]. Roughly 60% of patients have a defined genetic disease, with the majority of mutations mapping to genes encoding thick and thin myofilament proteins [3,4]. Of these defined loci, mutations in myosin heavy chain 7 (*MYH7*) and myosin binding protein C3 (*MYBC3*) are most common and are clinically characterized by asymmetric left ventricular thickening, diastolic dysfunction, and fibrosis [3]. For instance, the Arg403Gln (R403Q) mutation in the *MYH7* gene can result in a severe HCM phenotype with progressive myocardial dysfunction and increased incidence of sudden cardiac death [5,6]. Despite profound insights into the genetics of HCM, however, there is low correlation between mutation and clinical prognosis [7]. Moreover, there remains a need to define the earliest cell intrinsic responses to mutation prior to frank pathology.

Studies on isolated cardiac tissue have highlighted changes in contraction mechanics as potential drivers of the observed phenotype. HCM cardiomyocytes have increased tension costs (i.e., force generation per ATP utilized) due to changes at the biophysical level [8,9]. For example, both *MYBC3* and *MYH7* mutants exhibit a decreased number of super-relaxed-state myosin heads, a configuration with low ATPase cross-bridge utilization, and *MYH7* mutants also show faster cross-bridge kinetics that directly increase tension costs [8,10,11]. Moreover, HCM exhibits marked Ca^2+^ handling dysfunction (e.g., increased Ca^2+^ sensitivity and intracellular diastolic Ca^2+^), further exacerbating tension costs and ATPase activity [4,12]. Although the exact mechanism remains unclear, this high energy demand is hypothesized to increase mitochondrial workload and oxidative stress, ultimately resulting in maladaptive cardiac remodeling [13].

Insight into cardiomyocyte remodeling has come from large-scale molecular (i.e., omics-type) profiling studies, whose findings imply that increased energy demand damages mitochondria via augmented oxidative stress [9]. Mutant cardiac cells exhibit increased mitochondrial ultrastructure damage and evidence of oxidative damage (presumably resulting from increased reactive oxygen species generation) [14,15]. Excessive oxidative stress can impair mitochondrial components, such as the electron transport apparatus and mitochondrial DNA, which can exacerbate ATP generation deficits [9,16]. Notably, patient myectomies exhibit marked metabolic deficits associated with mitochondrial dysfunction, such as significantly decreased levels of fatty acid metabolic enzymes and lower ratio of phosphocreatine-to-ATP levels, which are presumably secondary to mitochondrial dysfunction [9,17,18].

Precision mass spectrometry is a powerful tool for elucidating biomolecular networks and associated signaling cascades driving pathobiology [19]. Recently, we applied a quantitative phosphoproteomic profiling workflow to study the impact of cardiac fibrosis in a three-dimensional biomimetic in vitro co-culture model system (heart-on-a-chip) and in myectomy specimens from HCM patients, which revealed pathway-level alterations associated with altered energetics and calcium handling [20]. However, this model lacked associated HCM mutations and major outstanding questions remained regarding molecular cascades driving mitochondrial dysfunction and associated excitation–contraction coupling perturbations.

To address these gaps, we have now performed a comparative multi-omics (proteomic, phosphoproteomic, and metabolomic) analysis to illuminate the early and direct consequences of mutations in *MYH7* in engineered human induced pluripotent stem-cell-derived cardiomyocytes (hiPSC-CM), in comparison to isogenic control cells, versus human HCM-patient-derived cardiac myectomy tissue, representing the spectrum of very early to clinically advanced stages of pathology. While some of the changes observed were co-ordinate, major differences were also detected. For instance, whereas we noted parallel increases in mitochondrial dynamics, favoring fission, in the early model and myectomy specimens, the myectomy specimens showed a complete abrogation of the major protective mechanisms against oxidative stress.

Integrated metabolic pathway analysis also highlighted increases in glutamine anaplerosis in the mutant hiPSC-CMs, which apparently replenished Krebs cycle intermediates and glutathione. Conversely, advanced-stage disease showed greatly diminished use of this pathway. We also characterized pronounced shifts in the post-translational (phosphorylation) states of the sarcoplasmic reticulum (SR) ATPase and functionally related excitation–contraction coupling proteins between the early and late disease stages, which correlated with alterations to Ca^2+^ handling, likely secondary to the metabolic defects.

In summary, our comprehensive assessment highlights metabolic, mitochondrial, and Ca^2+^ handling dysfunction across different stages of HCM pathology.

## 2. Results

### 2.1. Differential Phosphoproteomics in Early HCM Specimens

**Figure 1:**
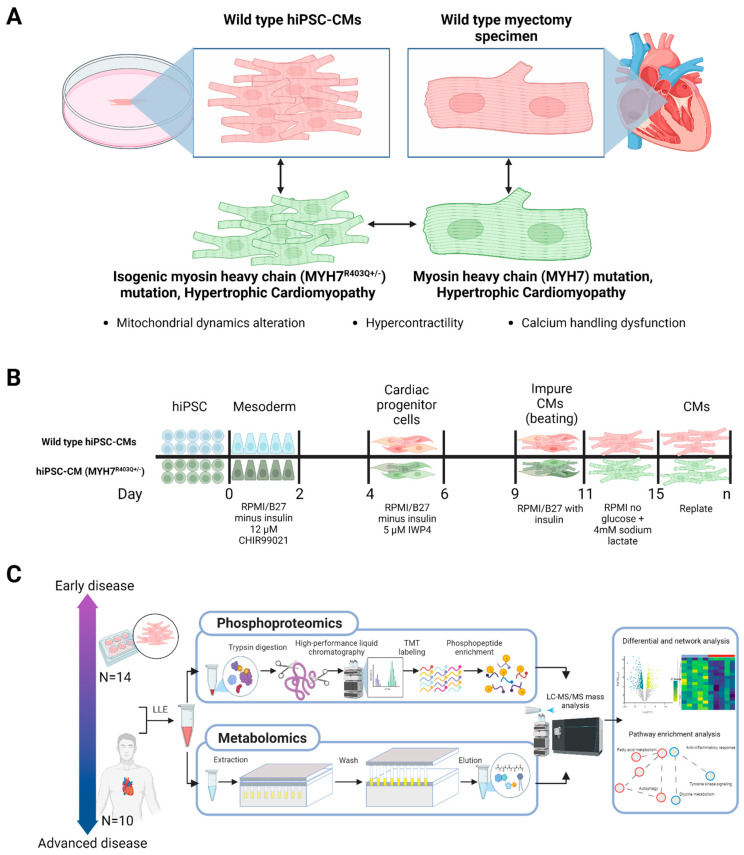
Study design and mass spectrometry workflows. (**A**) Schematic of wild-type and *MYH7* mutant human induced pluripotent stem-cell-derived cardiomyocyte (hiPSC-CM) and clinical (myectomy) specimens. (**B**) hiPSC-CMs differentiation protocol; cultures were differentiated over 20 days from CRISPR/Cas9-edited MYH7^R403Q+/−^ hiPSCs via targeted small-molecule, monolayer manipulation of the Wnt signaling pathway. (**C**) Sample processing followed by parallel mass spectrometry-based phosphoproteomics and metabolomics and subsequent bioinformatic analysis; LLE = liquid–liquid extraction, LC/MS = liquid chromatography/mass spectrometry, TMT = tandem mass tag.

To investigate metabolic status and mitochondria stress information in a model of the earliest pathobiological changes preceding overt disease, we first performed liquid chromatography–tandem mass spectrometry (LC/MS)-based global proteomics on 14 paired, isogenic hiPSC-CM samples (7 WT and 7 MYH7^R403Q+/−^ replicate cultures) (Figure 1A). The hiPSC-CM cultures were differentiated from CRISPR/Cas9-edited MYH7^R403Q+/−^ hiPSCs via small-molecule, monolayer manipulation of the Wnt signaling pathway (Figure 1B) [21,22]. MYH7^R403Q+/−^ mutations led to hypertrophy, heightened metabolic activity, and increased force output at the single-cell level [23]. To ensure quantitative accuracy, we subjected each proteolytically digested sample to stable isotope labeling using isobaric tandem mass tag reagents and augmented the depth of proteome coverage by applying extensive peptide-level offline prefractionation prior to precision Orbitrap mass analysis (see Section 4, Figure 1C) [24].

**Figure 2:**
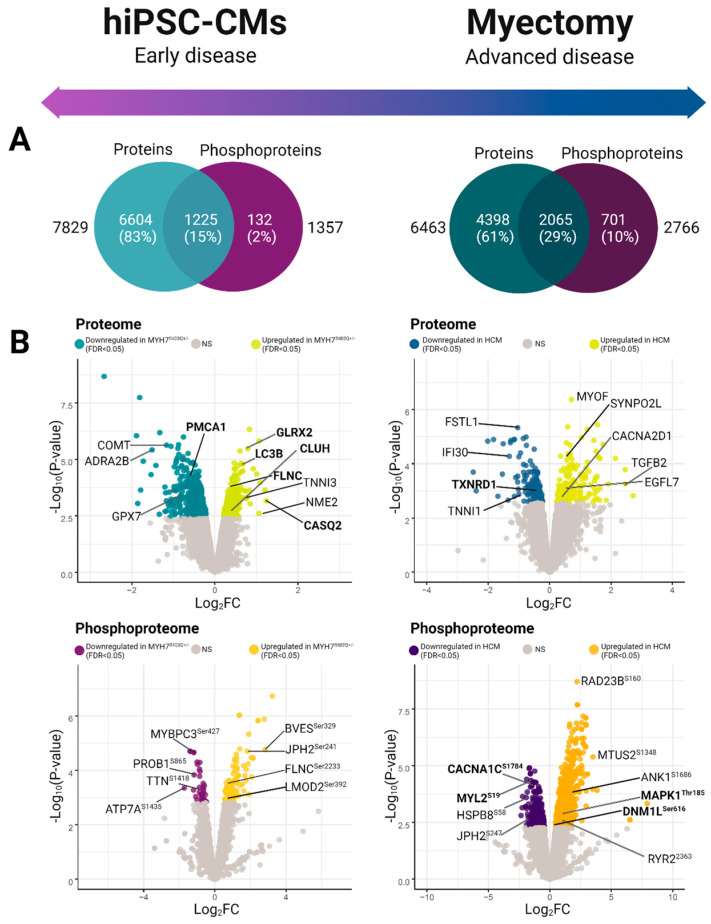
Global phosphoproteomic and proteomic expression profiles. (**A**) Venn diagrams depicting total feature coverage (high confidence proteins and phosphoproteins) quantified in hiPSC-CM (left) versus patient (hypertrophic cardiomyopathy specimens and healthy control) myectomy specimens (right). (**B**) Volcano plots of differentially expressed features. Plots show negative log-transformed *p*-values (moderated *t*-test) against log2-transformed fold-change difference measured between the mutant and wild-type specimens. Non-grey values highlight significant difference between groups (moderated *t*-test; FDR adjusted *p* < 0.05).

From our initial proteomic analysis, we identified and quantified 7829 proteins between the mutant and control (WT) hiPSC-CM samples (Figure 2A). Differential proteome analysis revealed 648 statistically significant proteins (moderated Student *t*-test, false discovery rate (FDR) adjusted *p* < 0.05) (Figure 2B, Supplementary Table S1A, Supplementary Figure S1) [25]. From this set, we captured differential components linked to myofibrillary function and stability, including elevated levels of troponin I (TNNI3), filamin-C (FLNC), and four and a half LIM domains protein 1 (FHL-1) [26,27]. Moreover, we observed significantly altered expression of factors involved in Ca^2+^ handling, including downregulation of L-type calcium channel subunits and upregulation of calsequestrin (CASQ2).

Since phospho-signaling responses provide additional functional information complementary to differential protein expression, we next performed large-scale phosphopeptide affinity capture and a secondary LC/MS analysis, which allowed us to map 4362 phosphorylation sites on 1357 distinct phosphoproteins in our hiPSC-CM model system (Supplementary Table S1B). We observed 129 statistically significant (FDR < 0.05) differential phosphosites (Figure 2A), which included altered phosphorylation patterns on key sarcomeric proteins, such as titin (TTN^Ser1423^), myosin-binding protein C (MYBPC3^Ser427^), filamin-C (FLNC^Ser2233^), and leiomodin-2 (LMOD2^Ser392^) (Figure 2B).

Collectively, these twin data sets offer a rich resource for analyzing biochemical processes altered in the mutant (early-stage) samples.

### 2.2. Pathway-Level Changes in Early Model of HCM Reveal Altered Mitochondrial Dynamics in Early HCM Specimens

**Figure 3:**
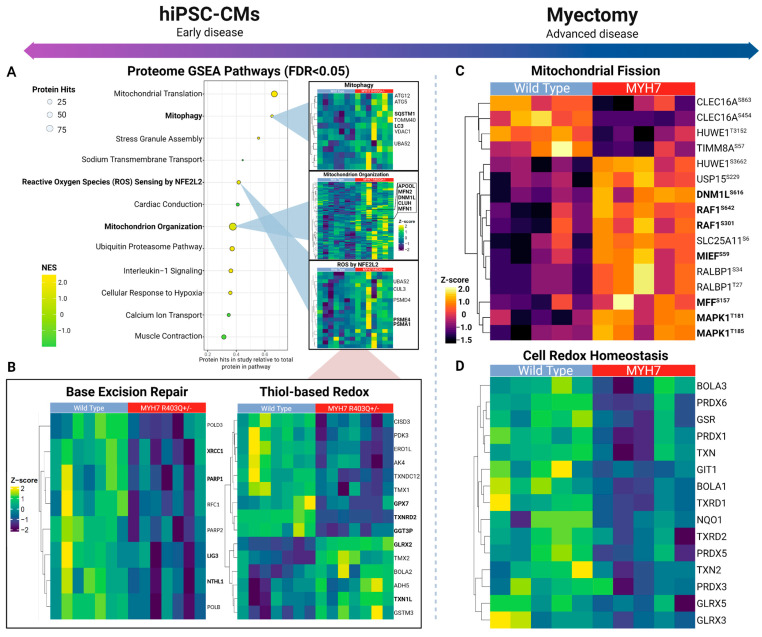
Mitochondrial dynamics across early and advanced specimens. (**A**) Top differential pathways from gene set enrichment analysis of mutant versus control hiPSC-CM proteome (left) with select heatmaps (right) showing hierarchically clustered (Euclidean distance) profiles of corresponding annotated protein components. (**B**) Depiction of base excision repair and glutathione-based redox proteins quantified in hiPSC-derived cardiomyocytes. (**C**) Mitochondrial fission-related phosphosites quantified in myectomy specimens. (**D**) Thiol-based antioxidant response enriched according to the affected patient proteome samples.

To systematically assess the pathway-level changes in the mutant cells, we performed gene set enrichment analysis (GSEA) with the proteomics data to parse annotated molecular pathways altered as a result of the MYH7^R403Q+/−^ mutation (Supplementary Table S2) [25,28]. We noted significant enrichment (FDR < 0.05) of the mitochondrion organization and mitophagy gene sets in the mutated cells (Figure 3A), which pointed to potential modulation of morphology and function. Within the mitochondrion organization pathway, increased expression of key mitochondrial fusion and fission effectors was detected. Most notably, fusion proteins upregulated in MYH7^R403Q+/−^ mutant cells included the primary GTPases driving fusion of the outer mitochondrial membranes, mitofusion-1 and -2 (MFN1 and −2) [29].

We also measured increased levels of Dynamin-1-like protein (DNM1L), the main GTPase conferring mitochondrial constriction during fission, as well as significant upregulation of clustered mitochondria protein homolog (CLUH) and mitochondrial Rho GTPase 2 (RHOT2), important regulators of mitochondrion trafficking that also mediate mitochondrial dynamics [29,30,31,32,33]. In particular, CLUH directly binds the mRNA transcripts encoding DNM1L receptor proteins (i.e., mitochondrial fission factor (MFF) and mitochondrial dynamics protein MID51 (MIEF1)) to facilitate fission [34,35]. Consistent with the upregulation of fission mediators, our phosphoproteomics data also showed an increasing trend (just short of statistical significance) in the phosphorylation levels of these DNM1L recruitment factors, MFF^Ser155^ and MIEF1^Ser55,59^ (Supplementary Table S1B).

Mitochondrial fission primes cells for mitophagy, a selective form of autophagy during oxidative stress, which fragments the mitochondrial network and degrades damaged mitochondria via autophagosome engulfment [36,37]. Consistent with this, we observed overexpression of a number of key modulators of mitophagy and autophagosome pathways in the mutant cells (Figure 3A), suggesting increased engagement of mitochondrial quality control mechanisms. Notably, microtubule associated protein 1 light chain 3 (MAP1LC3B) and its ubiquitin binding adaptor protein sequestosome-1 (SQSTM1) were upregulated, as were the related ubiquitin-like proteins ATG5 and -12 [38,39,40]. MAP1LC3B is important for maturation of the autophagosome, and the MAP1LC3B-SQSTM1 interaction is crucial for targeting mitochondria to autophagosome and initiating degradation [41].

Taken together, these findings provide evidence of increased fission and mitophagy in mutant CMs, highlighting these as potential determinants of mitochondrial health in early disease.

### 2.3. Insufficient Oxidative Stress Response Associated with Mutant hiPSC-CMs

Oxidative stress and mitochondrial damage are well-known features of cardiac disease [15,42]. In HCM, mitochondria are especially vulnerable to oxidative damage due to augmented reactive oxygen species (ROS) production [9,18,43,44]. An important consequence of oxidative stress is a change in mitochondrial morphology and function, including key macromolecule modifications [9,15]. Accordingly, we found the ROS sensing response as significantly enriched in the mutant hiPSC-CM samples (Figure 3A). This pathway includes components of the proteasome complex and assembly, which function in clearance of accumulated oxidized proteins, such as proteasome activator complex subunit 4 (PSME4) [45,46].

Moreover, we noted differential expression of thiol-based antioxidants, which protect against excessive ROS (Figure 3B). These included upregulation of components of the glutaredoxin and thioredoxin systems, which assist in reversing protein glutathionylation, a consequence of increased oxidative stress (i.e., cysteine residue thiol oxidation) (Supplementary Table S1A) [47,48]. In particular, we noted significantly (log_2_FC = 0.791) elevated expression of glutaredoxin-2 (GLRX2). However, we also detected decreases in other major components of these pathways, such as thioredoxin reductase 2 (TXNRD2) and glutathione peroxidase 7 (GPX7). TXNRD2 and GLRX2 serve similar roles in maintaining redox homeostasis by reducing mitochondrial-specific redox proteins, suggesting moderate antioxidant protection in the mutant hiPSC-CM [47].

Mitochondrial DNA is a principal target of oxidative stress (i.e., oxidized bases and strand breaks) [49,50]. Consistent with this, we noted significant decreases in key components of mitochondrial base excision repair (BER) (Figure 2B), the main repair mechanism of oxidative damage in the mitochondria [51,52]. For example, the mutant hiPSC-CM samples significantly downregulated endonuclease-III-like protein 1 (NTHL1), a bifunctional glycosylase that excises oxidized DNA bases and generates abasic sites [53]. We also noted decreases in Poly (ADP-ribose) polymerase 1 and 2 (PARP1/2), which detect abasic sites and recruit the DNA repair protein XRCC1 (XRCC1), forming a scaffolding complex for other repair factors [54,55]. While PARP1/2 are predominately localized to the nucleus, PARP1 migrates to the mitochondria via interactions with Mitofilin (IMMT), where it plays a protective/repair role for mtDNA by interacting with DNA ligase 3 (LIG3) [56,57]. Notably, then, is the observation that LIG3 also downregulated in our MYH7^R403Q+/−^ samples, which catalyzes the last ligation step of BER. Taken together, these data suggest that mtDNA is particularly vulnerable to oxidative damage, which could further facilitate mitochondrial dysfunction in mutant hiPSC-CMs.

### 2.4. Overlapping Mechanisms Driving Mitochondrial Dynamics and Downregulation of Oxidative Stress Response in Advanced HCM

To examine the persistence of the defects we observed in the mutant hiPSC-CM cells, we next measured the proteome and phosphoproteome of human donor biospecimens using the same workflow. We sampled explants from 10 sex-matched patients, 5 donor WT and 5 presenting with *MYH7* mutant alleles (Supplementary Table S3, Table 1). As with the hiPSC-CM model, we subjected the peptide samples to stable isotope labeling, followed by extensive prefractionation before in-depth quantitative LC/MS analysis. In total, we identified and quantified 6463 proteins along with 9697 phosphorylation sites on 2766 phosphoproteins in the human donor samples (Figure 2A).

Comparative statistical analysis demonstrated hundreds of statistically significant (FDR < 0.05) differences in proteins and phosphosites between the HCM patient and case controls (Figure 2A). Notably, we measured significantly elevated phosphorylation-based regulation of key mitochondrial fission factors via RAS/MEK/MAPK1 pathway activation (Figure 3C). For example, the HCM donors exhibited increased phosphorylation of serine-616 on Dynamin-1-like protein (DNM1L^Ser616^), which promotes activity and dimerization for constriction-based mitochondrial fission [58,59]. Further upstream, we noted evidence of activation of the mitogen-activated protein kinase (MAPK) cascade via hyperphosphorylation of the protein kinase domain on mitogen-activated protein kinase 1 (MAPK1^Thr185^), a conserved threonine/glutamate/tyrosine motif whose phosphorylation induces kinase activity [60]. MAPK1 increases mitochondrial dynamics by directly phosphorylating and activating DNM1L^Ser616^ [61,62]. Consistent with this, we detected significant phosphorylation of the mitochondrial outer membrane docking receptors for DNM1L, including MIEF^Ser59^, that are recruited during fission [34,63]. Hence, although not recapitulating the fusion and mitophagy alterations seen in the hiPSC-CM early model of pathology, these findings demonstrate a persistent upregulation of mitochondrial fission in advanced disease.

In contrast to the hiPSC-CM model, we observed profound decreases in factors linked to the oxidative stress response, such as thiol-based peroxidases (Figure 3D). The HCM patient samples exhibited significant decreases in the cell redox homeostasis pathway from GSEA, which included decreased expression of peroxiredoxin-6 (PRDX6) and mitochondrial thioredoxin (TXN2), important enzymes for reducing cellular peroxide levels (Supplementary Table S4, Supplementary Figure S2) [64]. From this pathway-level analysis, we detected decreased levels in thioredoxin reductase-1 and -2 (TXNRD1/2), which function to reduce thioredoxin, suggesting reduced thioredoxin antioxidant capacity [47,65]. Moreover, our analysis revealed concomitant alterations in key components of the double-strand DNA repair pathway (Supplementary Table S4), an important response that normally counters chronic oxidative stress [66].

Overall, in only partial accordance with the hiPSC-CM results, the advanced HCM specimens showed decreased expression in thiol-based oxidative response elements that are integral for maintaining mitochondrial integrity.

### 2.5. Integrated Metabolic Network Analysis Reveals Increased Dependence on Glutaminolysis in Early HCM

Metabolic remodeling is associated with increased mitochondrial dynamics in cardiomyocytes [67]. Thus, we applied an integrated metabolic pathway evaluation strategy on the hiPSC-CM samples to determine early metabolic alterations arising from the mutation.

**Figure 4:**
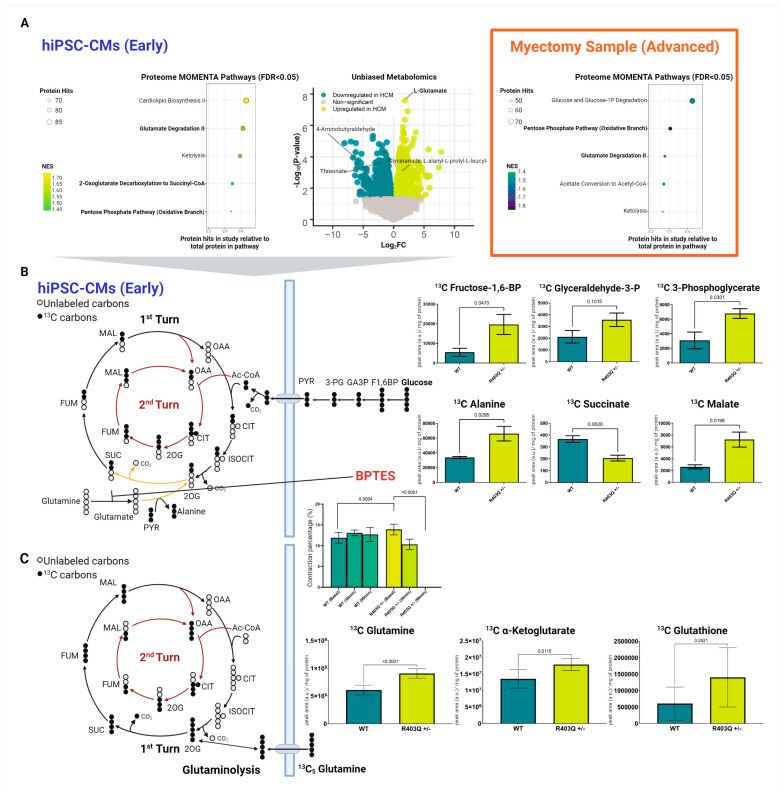
Integrated metabolomic analysis of patient myectomies and hiPSC-CMs. (**A**) Top differential pathways enriched in mutant versus control samples based on metabolic enrichment network analysis of the hiPSC-CM proteomic datasets (left), volcano plot of differential metabolite features from global (positive ion mode) metabolomic analysis of CM metabolites (middle), and enriched biochemical pathways revealed by metabolic enrichment network analysis of the patient myectomy samples (right). (**B**) Targeted mass-spectrometry-based metabolomics of hiPSC-CMs using isotopically heavy labeled ^13^C glucose, including after treatment with BPTES over a 90 min period. (**C**) Targeted mass-spectrometry-based metabolomics using isotopically labeled ^13^C glutamine. Individual data plots across all experiments are shown with means ± SD (*N* = 8). F1,6BP = fructose 1,6-bisphosphate, GA3P = glyceraldehyde 3-phosphate, 3-PG = 3-phosphoglycerate, PYR = pyruvate, Ac-CoA = acetyl-CoA, CIT = citrate, ISOCIT = isocitrate, 2OG = 2-oxoglutarate, SUC = succinate, FUM = fumarate, MAL = malate, OAA = oxaloacetate.

First, we performed metabolic enrichment network analysis (MOMENTA) to discover differentially expressed metabolic pathways predicted from our proteomic data, which we then validated by direct metabolomic analysis [68]. Among the many metabolic pathways altered in the early-stage mutant model (Figure 4A), we noted significant increases in enzyme levels mediating glutamate degradation, as well as 2-oxoglutarate (α-ketoglutarate) decarboxylation to succinyl-CoA, suggesting upregulation of glutaminolysis in the early HCM samples (Supplementary Table S5).

To confirm these predictions further, we performed two independent phases (global and targeted) of small-molecule mass-spectrometry-based metabolomics analysis to directly confirm changes in metabolic profiles and flux.

Consistent with our proteomics predictions, our global metabolomics survey found direct evidence for a significant increase in glutamate levels in the MYH7^R403Q+/−^ samples (Figure 4A, Supplementary Table S6). Among the hundreds of changes in metabolite levels that were detected, glutamate was among the top differential features (log_2_FC = 2.20, FDR < 0.001).

Given the evidence for glutaminolysis, we subsequently performed targeted metabolomic analyses to find further support of this pathway. For this analysis, we accurately quantified select glycolytic/citric acid cycle intermediates using ^13^C stable-isotope glucose tracer to determine whether unlabeled glutamate was supplementing the citric acid cycle (Figure 4B). As expected, the mutant cells showed elevated levels of labeled glucose-derived TCA metabolites, including ^13^C malate (Supplementary Table S7). In contrast, we observed a significant decrease in ^13^C succinate, the TCA produced following 2-oxoglutarate decarboxylation (Figure 4B). We additionally saw a significant increase in ^13^C alanine. Viewed from the vantage of current models of metabolism, these findings are consistent with the transamination of unlabeled glutamate (a downstream metabolite of glutaminolysis) with labeled pyruvate, resulting in increased labeled alanine and decreased succinate.

To verify that the enhanced glutamine is directly entering the citric acid cycle, we treated the WT and mutant hiPSC-CM cultures with isotopically (^13^C_5_) labeled glutamine (Supplementary Table S8). As expected, we observed a significant increase in labeled glutamine preferentially in the mutant cells (Figure 4C). Concomitantly, we detected increases in the labeled form of related TCA intermediate α-ketoglutarate in the HCM samples, further supporting the role of increased flux of glutamine into the TCA in the mutant cells.

To confirm the glutamine dependency of the MYH7^R403Q+/−^ cells, we treated the hiPSC-CMs with the selective glutaminase inhibitor BPTES. Strikingly, we saw a rapid and significant decrease in contraction over the treatment period in the mutant cells (Figure 4B), leading to a complete loss of contraction in the MYH7^R403Q+/−^ CMs by 90 min.

Intriguingly, we also detected a trend towards elevated glutathione-based synthesis from glutamine from our targeted metabolomics analysis (log_2_FC = 1.21), though slightly beyond statistical threshold (*p*-value = 0.0521) (Figure 4C, Supplementary Table S8). Our proteome data further supported this finding, as we observed significant decreases in glutathione catabolic components (Figure 3B). These included the putative catabolic factor gamma-glutamyl transpeptidase 3 (GGT3P). Moreover, MOMENTA revealed enrichment in the mutant cells of the pentose phosphate pathway, which produces NADPH (Figure 4A). NADPH is a cofactor for glutathione reductase that enables reduction of glutathione to alleviate oxidative stress [69]. We likewise detected increased levels of the fructose-2,6-bisphosphatase enzyme TIGAR, which increases the activity of the pentose phosphate pathway [70,71]. Glutathione is the primary intracellular thiol-based redox buffer, further supporting the protective engagement of the thiol-based oxidative stress response machinery in the early-stage disease model [48].

Taken together, our comprehensive metabolic analysis of the hiPSC-CM model strongly supports glutamine anaplerosis as an important mechanism for replenishing TCA metabolites as an early adaptive response to altered contractile function.

### 2.6. Metabolic Network Analysis Reveals Decompensated Metabolism in Advanced Specimens

In striking contrast to the results from our cell culture model, our proteomics analysis of the myectomy samples indicated decreased metabolic supplementation through the TCA/glutamine shunt.

Beyond significant reductions in the components of the glutamate degradation II pathway from MOMENTA (Supplementary Table S9), our GSEA of the clinical samples also revealed a significant (FDR < 0.05) decrease in mitochondrial fatty acid beta-oxidation and citric acid cycle/respiratory electron transport gene sets in the affected patient specimens (Figure 4A, Supplementary Table S4). Within the citric acid cycle/respiratory electron transport set, the HCM samples showed markedly decreased expression of electron transport complex proteins, such as NADH dehydrogenase (ubiquinone) 1 (NDUFV1).

Moreover, we noted decreases in several enzymes crucial in fatty acid metabolism, such as ACAT1 (acetyl-CoA acetyltransferase) and ACAA2 (3-ketoacyl-CoA thiolase) from the fatty acid beta-oxidation gene set (Supplementary Table S4). Given the importance of fatty acid oxidation in cardiac cells, these findings suggest poor metabolic capacity in the affected tissue [72].

Lastly, again in contrast to our early model, we noted significantly decreased enrichment of the pentose phosphate pathway in the advanced disease with respect to myectomy controls (Figure 4A). This pathway is crucial for its production of NADPH, which is used by glutaredoxin and thioredoxin systems to reduce key antioxidant enzymes.

Taken together with the decreases seen in mitochondrial-specific metabolic pathways, compounded with the decrease in glutamine anaplerosis, these findings demonstrate the potential for the persistence in impaired bioenergetic replenishment and antioxidant protection in advanced HCM.

### 2.7. Phosphorylation-Dependent Regulation of Excitation Contraction Coupling in hiPSC-CMs

Given the profound differences in mitochondrial function and metabolism noted in both the mutant model and advanced HCM specimens, we anticipated persistent dysregulation of calcium handling and contractility mechanisms in both contexts.

**Figure 5:**
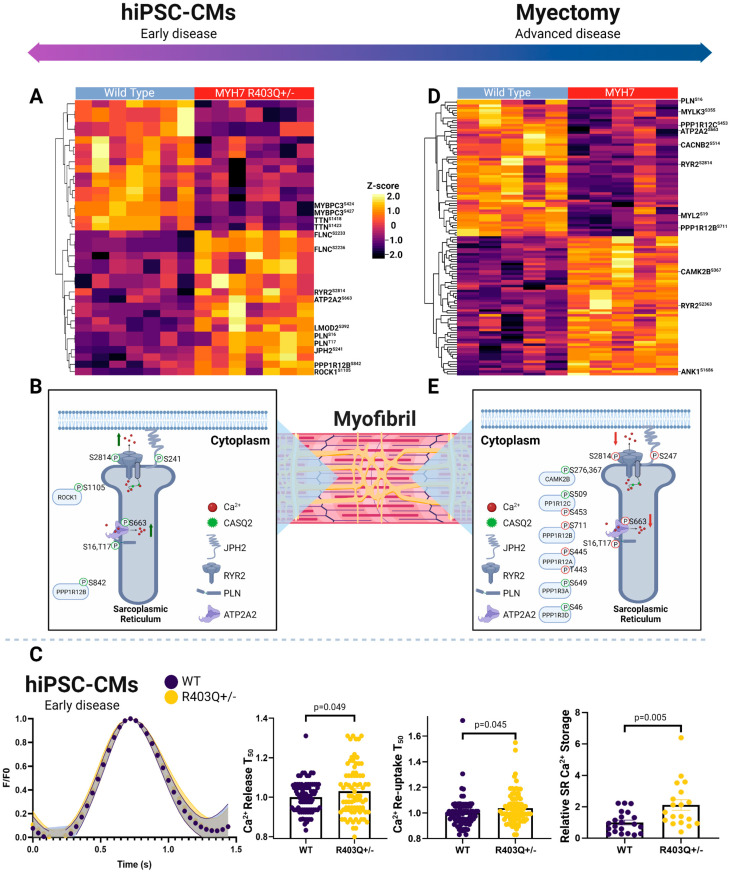
Phosphoproteomic landscape of excitation contraction coupling proteins. (**A**) Hierarchical clustering (Euclidean distance) showing changes in phosphosite abundance of significantly differentially phosphorylated proteins linked to excitation contraction coupling in hiPSC-CMs and (**B**) patient myectomy samples. (**C**) Schematic depiction of select SR phosphoproteins in hiPSC-CMs and (**D**) clinical specimens. (**E**) Plots showing average Ca^2+^ transients (*N* = 3; n > 75 cells) normalized to WT transient, with error bands representing SEM (left), relative time for Ca^2+^ release into and reuptake from the cytoplasm compared to average WT cell (*N* = 3; n > 83 cells) (middle), and relative intensity of Ca^2+^ release into cytoplasm from SR following caffeine bolus relative to the average WT cell (right). Individual data points (n) across all experiments (*N*) are shown with means ± SEM.

First, examining the hiPSC-CM model, we observed significantly altered phosphosites on key calcium handling factors localizing to the sarcoplasmic reticulum, mitochondria, sarcomere, and plasma membrane between the mutant and control samples in both the early model and advanced disease stages. For instance, in the hiPSC-CM mutant cells, we measured significantly increased phosphorylation events on a number of key SR proteins (Figure 5A, Supplementary Table S1B). These included hyperphosphorylation of cardiac phospholamban (PLN^Ser16,Thr17^), ryanodine receptor 2 (RYR2^Ser2814^), sarcoplasmic/endoplasmic reticulum calcium ATPase 2 (ATP2A2^Ser663^), and junctophilin-2 (JPH2^Ser241^). The RYR2^Ser2814^ and PLN^Ser16,Thr17^ events are linked to increased flux of calcium through the SR, with RYR2^Ser2814^ associated with channel opening and augmented outward flux, while PLN^Ser16,Thr17^ has been shown to increase inward flux by releasing PLN inhibition of ATP2A2 (Supplementary Table S10A) [73,74].

We additionally measured persistent changes in the regulation of key sarcomeric proteins modulating contractility. These included increased phosphorylation of filamin-C (FLNC^Ser2233,2236^) and leiomodin-2 (LMOD2^Ser392^), along with decreased levels of major phosphosites on titin (TTN^Ser1423,1418^) and myosin-binding protein C (MYBPC3^Ser424,427^). While M-domain phosphorylation on MYBPC3 is crucial for modulating the ATPase activity of myosin via stability of its super relaxed state and its interaction with F-actin, we captured events in the adjacent C2 domain, an F-actin binding domain [75,76]. Given the decrease in super-relaxed-state myosin heads, this event could represent a novel mode of phosphorylation events increased in HCM regulation for this process and energetics. We also noted increased phosphorylation LMOD2, another actin binding protein that regulates contractility, and the protein phosphatase 1 regulatory subunit 12B (PPP1R12B^Ser842^) and rho-associated protein kinase 1 (ROCK1^Ser1105^), upstream modulators of calcium sensitivity of the contractile machinery (Figure 5B) [77,78,79].

From our proteomics analysis of the early disease model, we observed decreases in the abundance of key plasma membrane calcium channels, as well as electrochemical gradient-forming ATPases (Supplementary Table S1A). These included decreased expression of the β-2/-3 and α2/δ3 subunits of the L-type calcium channel and plasma membrane calcium-transporting ATPase 1 (PMCA1), which directly modulate calcium flux at the plasma membrane [80,81]. Moreover, the MYH7^R403Q+/−^ hiPSC-CM samples had decreased levels of the sodium/potassium-transporting ATPase subunit alpha-1/2 (catalytic) and beta-1/2, which are essential for cardiac excitability by assisting in calcium extrusion at the membrane [82]. Furthermore, they displayed reductions in CASQ2 and cardiac junction (ASPH), which play key roles in SR Ca^2+^ storage and the magnitude of release during excitation-contraction coupling buffering via interacting with ryanodine channels [83,84].

Overall, these findings suggested increased SR Ca^2+^ storage, with concurrent increases in Ca^2+^ flux throughout the SR in the MYH7^R403Q+/−^ cells. To independently validate our prediction of defects in Ca^2+^ handling in the mutant cells, we directly measured Ca^2+^ flux in paced hiPSC-CMs via live-cell Ca^2+^ imaging. As summarized in Figure 5C, we observed a significant increase in SR Ca^2+^ storage in the HCM cells relative to WT following caffeine treatment, in line with our proteome measurements of CASQ2 expression. Surprisingly, however, we also detected a significant increase in time of Ca^2+^ release and re-uptake into the SR, despite the increased flux predicted from our phosphoproteome analysis. We note that this counterintuitive result is potentially explained by the increase in overall SR Ca^2+,^ by which the additional Ca^2+^ must be pumped against a higher concentration gradient, thus increasing ATP2A2 (SERCA) energetic requirements. This would be expected to exacerbate ATP production demands on the mitochondria and to further thermodynamically limit SERCA function [85].

### 2.8. Phosphorylation-Dependent Regulation of Excitation Contraction Coupling in Myectomy Specimens

Focusing on pronounced changes in the HCM tissue samples, we again consistently measured differential phosphosites belonging to the excitation contraction coupling mechanism in advanced disease, as was seen in the early-stage model, though we noted a markedly different phosphorylation landscape (Figure 5D, Supplementary Table S3B). Top differential excitation contraction coupling phosphosites included downregulation of RYR2^Ser2811^ and JPH2^Ser247^ and increases in RYR2^Ser2363^ AKAP-12^Ser283,286,T285^/-13^Ser2398,2728^, ankyrin-1 (ANK1^Ser1686^). Yet, in contrast to the early HCM model, we detected decreases in PLN^Ser16,Thr17^, RYR2^Ser2814^, and ATP2A2^Ser663^, which were increased in the early disease model samples (Figure 5E, Supplementary Table S10B).

We also noted increases in effectors that directly phosphorylate SR ATPases (e.g., RYR2), calcium channels, and sarcomere proteins to modulate Ca^2+^ homeostasis and sensitivity, which dictate overall ATP utilization. For example, protein phosphatase 1 and its regulatory subunits decrease PLN^Thr17^ phosphorylation, allowing PLN to increase its inhibitory effects on ATP2A2 [74]. In the HCM tissue, we captured differential phosphorylation on regulatory subunits PPP1R12B^Ser711^, PPP1R12C^Ser509, 453^, PPP1R12A^Thr443, Ser445^, PPP1R3A^Ser649^, and PPP1R3D^Ser46^. Other effectors seen in the clinical specimens included key upstream modulators of Na^+^ and Ca^2+^ homeostasis regulation, such as calcium/calmodulin-dependent protein kinase type II subunit beta (CAMK2B^Ser367,276^).

Other important regulators of sarcomeric calcium sensitivity showing persistent changes in advanced disease included differential phosphorylation of TTN, FLNC, synaptopodin-2 (SYNPO2), MYBPC3, and myosin regulatory light chain 2 (MYL2) (Figure 5D). In particular, the MYL2^Ser15,19,Thr24^ decreased phosphorylation levels in advanced HCM, as well as decreased phosphorylation of its upstream kinase MYLK3^Ser152,355,Thr359^, having important regulatory implications, since these phosphosites function in cross-bridge kinetics [86]. For instance, the serine-15 modification increases lever arm stiffness and myosin attachment, enhancing contraction and protecting against hypertrophy-related stress [87,88].

Among the main plasma membrane channels detected in the clinical samples, we found decreased phosphorylation of voltage-dependent L-type calcium channel subunits (CACNB2^Ser514,550^ and CACNA1C^Ser1784^) and potassium voltage-gated channels (KCNH2^Ser255^) in the HCM tissue. These components regulate the influx of calcium into the cytoplasm and the rectifying potassium current, respectively, and their phosphorylation provides precise regulation of open probability [89].

The overall phospho-pattern of SR proteins and modulators of contractility suggests differing signaling-based regulation in advanced disease compared to the early specimens.

## 3. Discussion

While previous molecular studies by our group and others have elucidated important mechanisms of HCM pathobiology, few have explored consequences associated with MYH7 mutations over different disease-state phenotypes [9,90,91]. Through an integrated quantitative multi-omics analysis, we captured hundreds of additional differential features which mapped to distinct molecular mechanisms dictating mitochondrial homeostasis in both early and advanced models of HCM, as well as stage-specific metabolic pathways and excitation-coupling mechanistic adaptations.

Although there are well-known limitations associated with hiPSC-based models, in particular the immature fetal-like maturation states, our mutant system provides a tractable means to explore the direct molecular and phenotypic consequences of defective MYH7 function at the early stages of pathology. Conversely, despite substantial clinical heterogeneity in HCM presentation, our comparative analysis of patient myectomy specimens, belonging to a single defined mutation subpopulation, allowed us to elucidate perturbations associated with both early and late stages of pathology. Moreover, while we analyzed an array of MYH7 mutations in this subpopulation, we were able to identify consistent findings amongst late-stage HCM specimens.

Oxidative stress and mitochondrial damage are commonly described features of HCM [9,18]. In physiological conditions, the deleterious effects of ROS (e.g., oxidative modification) are balanced by endogenous antioxidant systems [92]. However, excess production can impair the function of mitochondrial components [5,93,94]. We observed a mixed antioxidant response in the mutant hiPSC-CM, followed by a near total loss in this pathway signature in the advanced myectomy specimens. The early-stage in vitro model suggests increased protection against protein glutathionylation through thiol-based systems, the primary intracellular redox buffer for protecting protein cysteine residues from oxidative modification. We found evidence in both our proteomics and metabolomics data for increased antioxidant protection via expression of glutaredoxin and thioredoxin enzymes and upregulation of the pentose phosphate pathway. The latter provides NADPH for reducing oxidized glutathione, allowing another redox cycle [95]. From our targeted metabolomics experiments, we confirmed that glutamine moved faster towards GSH synthesis in the mutant cells, consistent with a crucial role of glutaminolysis for cardiomyocytes under oxidative stress [96]. Thus, there appears to be a relatively robust intracellular antioxidant protection early on, which likely serves to counteract the known overproduction of ROS reported before in in vitro HCM models [14,15].

Conversely, our myectomy data revealed a profound loss of these protective mechanisms in advanced stage disease, including TXN2 and its reductase TXRN2. These expression deficits are likely associated with the reduced ROS scavenging capabilities noted by previous studies of HCM myectomy samples, particularly the increased ratio of GSSG:GSH (i.e., reduced GSH antioxidant capacity) [15,97].

Interestingly, Wang et al. recently reported increased evidence of pentose phosphate pathway in advanced HCM, but our own patient cohort data suggest the opposite trend [90]. In contrast to their KEGG pathway analysis, our integrative MOMENTA framework detected significant downregulation of transaldolase and the NAD-dependent master regulatory deacetylase enzyme sirtuin-2. One potential explanation for this discrepancy is the clinical heterogeneity of HCM, which varies greatly from mutation to mutation. While we exclusively utilized MYH7 mutants, Wang et al. analyzed samples representing an array of mutations. Overall, our observation of poor thiol-based antioxidant protection in the clinical HCM specimens suggests exacerbation of the mitochondrial damage and dysfunction observed before in myectomy specimens [9].

Given the importance of double-strand break response in oxidative stress, the decreased expression of DNA repair mechanisms in our analysis of advanced HCM specimens further demonstrates poor oxidative stress adaptation [66]. Mitochondrial DNA is especially vulnerable to oxidative modification given its proximity to ROS production [98]. Hence, the decreased expression of BER enzymes in the mutant hiPSC-CM model implies the potential accumulation of damaged mtDNA at the early stages of pathology. Mitochondrial damage is known to result in reduced bioenergetics and could be the cause of the overall decreased ATP generation and mitochondrial metabolic pathways noted before in advanced HCM [9,99]. Further supporting this, a number of cardiomyopathies linked to mtDNA mutations exhibit pronounced mitochondrial dysfunction [100].

Mitochondrial dynamics are an important mechanism for maintaining mitochondrial homeostasis and function [23,101]. Mitochondrial fusion ensures optimal respiratory function by exchanging contents (e.g., proteins and DNA) after merging [102,103]. Conversely, fission is the division of a single mitochondrion into two daughter organelles, which assists in mitochondrial distribution but can also serve in quality control during oxidative stress via activation of mitophagy. Imbalances between these processes can result in malfunctioning mitochondria and metabolic disturbances [101]. There is substantial evidence that fission and fusion processes are active in HCM pathology, especially given the increases in mitochondrial number and cristae disorganization noted before in both clinical and in vitro culture models of HCM [9,23]. Accordingly, we noted an increase in mitochondrial dynamics in response to mutation in both the early and advanced pathology. In our advanced specimens, we found significant phosphorylation of DNM1L^Ser616^ and its recruitment factors MFF^Ser157^ and MIEF^Ser59^, which are the principal components for fission [104]. This echoes the increased evidence from Ranjbarvaziri et al., in which they found this ultrastructural mitochondrial remodeling from imaging [9]. Perplexingly, despite increased oxidative stress (e.g., elevated 4-hydroxy-2-nonenal-modified proteins) and the mitochondrial damage noted in their study, they failed to find molecular activation of the fusion/fission mechanisms. In contrast, we provide direct phosphorylation level regulation of this pathway, which helps explain those ultrastructural changes in HCM.

Though we found that the regulators of fission and fusion were upregulated in both early and advanced stages, we only found direct evidence of mitophagy in the in vitro culture model. Mitophagy is important for clearing impaired mitochondria via the autophagosome–lysosome pathway following fission/fusion and would provide another means of combating oxidative stress [105]. We failed to find direct molecular evidence to support elevated mitophagy in advanced specimens. Mitochondrial dynamics and mitophagy are dependent on cardiolipin lipid species for recruiting fusion and fission mediators [106]. Consistent with this, our MOMENTA of the hiPSC-CM samples showed increased enrichment of cardiolipin biosynthesis in the mutant cells (Figure 4A), as well as increased expression of cardiolipin synthase (CRLS1), which catalyzes the last step in de novo cardiolipin synthesis (Supplementary Table S1A). In contrast, the advanced specimens showed decreased expression of CRLS1 and the lysocardiolipin acyltransferase 1 (LCLAT1) (Supplementary Table S3A), both crucial enzymes for cardiolipin production in vivo [107]. Given the previously reported decreases in cardiolipin species in advanced HCM, the failure to upregulate these mechanisms could be associated with failure to upregulate mitophagy in advanced specimens [9].

The metabolism of cardiomyocytes is dynamic and cardiac tissues are able to adapt their preferences for carbon sources depending on metabolic demand [96,101,108]. Our discovery of increased glutamine anaplerosis in early mutant samples is one such demonstration, noting that glutamine catabolism may serve as an important contributor to preservation of cardiac function in early-stage pathology. We demonstrated that increased glutaminolysis serves a biosynthetic role in the mutant CMs, replenishing the TCA intermediate 2-oxoglutarate via transamination of glutamate, as well as providing a major precursor for glutathione synthesis. In contrast, the advanced samples exhibited decreased glutamate degradation, a pathway downstream of glutamine anaplerosis. Recently, Watanabe et al. found that glutaminolysis improves cell viability in healthy cardiomyocytes under oxidative stress [96]. We provide compelling evidence that this mechanism provides a dual protective role in HCM, while its loss in advanced stage disease likely represents a further maladaptation to chronic oxidative stress.

Excitation contraction coupling, particularly ATPase-dependent components, rely on readily available ATP for contiguous function. Unsurprisingly, our global proteome and phosphorylation landscape captured distinct calcium handling and calcium sensitivity between HCM and normal controls in both the in vitro and in vivo contexts. Our analysis of the mutant cell cultures, which showed increased metabolic activity supplemented by glutaminolysis, revealed significant activation of SR calcium handling proteins in HCM. We noted increases in PLN^Ser16,Thr17^, events that remove its reversible inhibition of ATP2A2 [74]. Importantly, these sites are regulated by distinct kinases (e.g., CAMK2A vs. PKA), demonstrating multiple levels of regulation. Since this latter enzyme accounts for the majority of Ca^2+^ reuptake, reducing the degree of inhibition is predicted to increase SR Ca^2+^ reuptake activity. This regulation is likely necessary given the increased SR Ca^2+^ storage in MYH7^R403+/−^ cells, revealed from Ca^2+^ imaging, particularly when coupled to the decrease we observed in PMCA1 and supporting Na^+^/K^+^ ATPases, which assist ATP2A2 in decreasing cytosolic Ca^2+^ for relaxation. This high Ca^2+^ reuptake burden on SR ATPases is expected to enhance ATP demands over time, and may be a predominant initial source of mitochondrial ATP demand. Conversely, we noted an opposite trend on SR handling in the advanced disease samples, with decreased phosphorylation events at the same sites. This suggests a lower burden of ATPase-dependent activity, perhaps due to decompensated ATP production. Other novel findings included phospho-regulation of contractility, such as decreased MYBPC3 phosphorylation in both systems.

Collectively, this study fills in gaps from previous studies of HCM and expands knowledge of the initial responses to mutations that protect against early-stage cardiac pathology that eventually are overwhelmed, leading to irreversible advanced disease. Beyond the molecular insights highlighted, all the data are being made publicly available to serve as a community resource for future mechanistic studies.

## 4. Materials and Methods

Human induced pluripotent stem cell (hiPSC)-derived cardiomyocyte cell culture—Human iPSC-derived cardiomyocytes were generated from the Harvard Personal Genome Project line 1 (PGP1, GM23338), generously provided by the Seidman Lab at Harvard Medical School [23,109]. Heterozygous mutations were introduced into the *MYH7* allele in hiPSC as described [23,110]. hiPSCs were cultured with mTESR1 media (STEMCELL Technologies, Vancouver, BC, Canada) on Matrigel (Thermo Fisher Scientific, Waltham, MA, USA)-coated plates and differentiated into cardiomyocytes (day 0) at 80–100% confluency via activation of the WNT pathway with 12 μM CHIR 99021 (Tocris, Bristol, UK) in RPMI + GlutaMAX media supplemented with B27 minus insulin (RPMI and B27 minus, Thermo Fisher Scientific). After 24 h, the cells were washed with PBS and given RPMI and B27 minus. On day 3, the WNT pathway was inhibited via 5 μM IWP-4 (Tocris) in fresh RPMI and B27 minus media for 48 h. On day 5, the media was replaced with fresh RPMI and B27 minus and changed every other day with the use of B27 plus insulin, starting on day 9. On day 11 and 13, the cardiomyocyte population was purified by metabolic selection via RPMI glucose-free media (Gibco, Waltham, MA, USA) supplemented with 4 mM of DL-lactate (MilliporeSigma, Burlington, MA, USA). On day 15, the media was replaced with RPMI + GlutaMAX media supplemented with B27 plus insulin. Following 48 h, cells were replated onto fibronectin-coated tissue culture plastic plates using 0.25% Trypsin-EDTA (Thermo Fisher Scientific) and 10 μg/mL deoxyribonuclease I (STEMCELL Technologies). They were then placed in RPMI B27+ supplemented with 5 μM Y-27632 (Tocris) and 2% fetal bovine serum (MilliporeSigma) for seeding. Cultures were maintained on RPMI + GlutaMAX media supplemented with B27 plus insulin until harvesting at 30+ days post-differentiation. For harvesting, cells were trypsinized, centrifuged, and snap frozen in liquid nitrogen for proteomics analysis.

Patient Sample Acquisition—Surgical myectomy tissue was obtained from HCM patients with symptomatic LVOT obstruction. Patients gave informed consent for their myectomy tissue to be used in research. *MYH7* pathogenic variants were identified by whole exome sequencing of peripheral blood mononuclear cell DNA and confirmed by commercially available Gene Panels (GeneDx, Stamford, CT, USA). Myectomy sample processing was conducted as previously described [111,112,113,114]. A total of 100 mg of collected myectomy tissue was minced into 1 mm^3^ pieces, placed in 0.5 mL of CryoStor CS10 Freeze Media (STEMCELL Technologies), and stored in a MrFrosty (ThermoFisher) at 4 °C for 10 min, and then transferred to −80 °C overnight. Sample collection was approved by the Tufts University/Medical Center Health Sciences Institutional Review Board under IRB protocol # 9487. All subjects gave their informed consent for inclusion before they participated in the study. The study was conducted in accordance with the Declaration of Helsinki. Tissue from organ donor patients without underlying cardiac disease was obtained and processed as described previously [111,112,113,114].

Homogenization, Metabolite/Protein Extraction, and Trypsin Digestion—The workflow is summarized in Figure 1C. For hiPSC-CMs, frozen samples were resuspended in a 4 vol. mixture of ice-cold methanol/acetonitrile/water (MeOH/ACN/H2O, 40/40/20, *v*/*v*; MS-grade, Fisher) in chemically resistant microcentrifuge tubes (Eppendorf, Hamburg, DEU). The samples were flash frozen in liquid nitrogen for 1 min, allowed to thaw, and sonicated on ice (Ultra Autosonic, Pune, IND) for 5 min three times. The extracts were then incubated at −20 °C for 1h and centrifuged at 12,000× *g* at 4 °C for 15 min to pellet the protein precipitate. The supernatants (with the metabolites) were transferred to new microtubes, dried under vacuum at 30 °C. The crude metabolite extracts were then subjected to solid-phase micro-extraction (SPME) for cleaning up. In brief, the coated SPME blades were preconditioned in MeOH/H2O (50:50, *v/v*) for 30 min. The samples were resuspended in 2% MeOH and incubated for 1 h with the resins (blades). After the blades were rinsed for 20s using water, the bound metabolites were desorbed using ACN/H2O (50:50, *v/v*) for 1 h. The eluted metabolites were dried under speedvac (Eppendorf) and kept at −80 °C prior to LC-MS analysis. The protein precipitate was transferred to 200 µL of lysis buffer containing 6 M GuHCl, 100 mM Tris pH 8.5, 1 mM CaCl2, 10 mM TCEP, and 40 mM CAM, with EDTA-free protease inhibitor (Sigma) and phosphatase inhibitor (PhosSTOP Roche, MilliporeSigma), and heated for 6 min at 95 °C. Human samples were placed in 5 vol. lysis buffer containing 8M urea, 5mM dithiothreitol (DTT), 50 mM ammonium bicarbonate (NH4HCO3), with EDTA free protease inhibitor (Sigma), and phosphatase inhibitor (PhosSTOP Roche). Afterwards, they were mechanically homogenized. All samples were sonicated on ice (Branson, Brookfield, CT, USA). Protein quantity was assessed by Bradford protein assay (Bio-Rad, Hercules, CA, USA), followed by digestion overnight at 37 °C with sequencing-grade Trypsin (1:50 enzyme to protein ratio, w/w, ThermoFisher). After adding trifluoroacetic acid to 0.1% *v/v*, peptide digests were desalted using a C18 Sep-Pak (Waters, Milford, MA, USA) according to the manufacturer’s instructions, resuspended in 100 mM TEAB, and quantified by Quantitative Colorimetric Peptide Assay (Pierce) prior to TMT labeling.

Stable-Isotope Labeling and Offline Reverse-Phase High-Performance Liquid Chromatography Fractionation—For each sample, 100 µg of peptide digest (adjusted to 100 µL with 100mM TEAB) was mixed with a unique amine-reactive isotope-coded isobaric tandem mass tag (TMT-16-plex) reagent (Thermo Fisher Scientific) prior to sample multiplexing and precise quantification by LC/MS. After pooling, labeled peptide was desalted, dried, and suspended in 300 µL buffer containing 0.1% ammonium hydroxide and 2% acetonitrile (ACN). The pooled sample mixture was pre-fractionated by high pH reverse-phase HPLC on a XBridge Peptide BEH C18 column (130Å, 3.5 μm, 4.6 mm × 250 mm, Waters) using an Agilent 1100 HPLC system. Peptides were eluted using a gradient of mobile phase A (0.1% NH4OH −2% ACN) to B (0.1% NH4OH −98% ACN) over 48 min and collected as 12 pooled fractions. For phosphoproteomics, the bulk (95%) of each sample was subject to phospho-peptide enrichment using FeO_2_ metal-chelate resin (PureCube Fe-NTA MagBeads, Cube Biotech, Rhein, DEU) [24], while the remaining (5%) portions were analyzed directly by nanoflow LC/MS as bulk proteome measurements (a total of 24 injections, 12 for proteomics and 12 for phosphoproteomics).

Mass Spectrometry Analysis of Peptides and Identification—Isotope-labeled peptides were reconstituted in mobile phase A (0.1% formic acid, 2% ACN) prior to LC/MS analysis on a Thermo-Fisher Exploris 480 hybrid quadrupole-Orbitrap mass spectrometer interfaced with Thermo-Fisher FAIMS Pro with integrated Proxeon EASY-nLC 1200 system. After loading onto a C18 reverse-phase pre-column (75 μm i.d. × 2 cm, 3 μm, 100Å, Thermo Fisher Scientific), peptides were gradient separated on an EASY-Spray C18 nanocolumn (75 μm i.d. × 50 cm, 2 μm, 100Å; ES803A, Thermo Fisher Scientific) using 2–35% mobile phase B (0.1% formic acid, 80% ACN) over 120 min (proteome) or 180 min (phosphoproteome), and electro-sprayed at ~250 nL/min into the Exploris instrument operated in positive ion mode (capillary temperature 275 °C, 2100 V potential). Data-dependent spectra were acquired automatically via high-resolution (60,000) precursor ion scan (350–1500 *m*/*z* range) to select the 12 most intense peptides for MS/MS fragmentation by high energy dissociation (normalized collision energy of 33 at 45,000 resolution).

The resulting RAW files were searched by MaxQuant (1.6.7.0) using default settings against the human proteome (SwissProt Taxonomy ID: 9606, downloaded September, 2021), allowing for two missed cleavage sites and variable modifications (Ser/Thr/Tyr phosphorylation, N-terminal acetylation, and Met oxidation) and carbamidomethylation of cysteine and TMT labels as a fixed modification. Peptide- and protein-level matches were filtered to high confidence (1% FDR), with a minimum phosphosite localization probability of 0.7. TMT quantification involved label correction (lot values provided by ThermoFisher).

Phosphoproteomics Statistical Analysis and Pathway Enrichment—Bioinformatic analysis was performed using R (language and environment for Statistical Computing; http://www.R-project.org, accessed on 12 May 2021.). Peptide feature intensities were log transformed and loess normalized. LIMMA R package was used for differential analysis (moderated Student *t*-tests), and to generate ranked lists for subsequent enrichment analysis using the Benjamini–Hochberg FDR correction [25,115]. Statistical enrichment analysis was performed using fgsea R package [28,68]. Volcano plots were created with the EnhancedVolcano R package [116]. All figures were created with BioRender.com.

Mass Spectrometry of Metabolites and Identification—Metabolites were reconstituted in mobile phase A (2% ACN) prior to metabolite nanoflow (nLC) LC/MS analysis on a Hybrid Quadrupole-Orbitrap Q-Exactive HF (Thermo Scientific). After loading onto a C18 reverse-phase pre-column (75 mm i.d. × 2 cm, 3μm, ThermoScientific), metabolites were separated on a capillary column (75 mm i.d. × 25 cm, 2 μm, 100 Å, ThermoFisher Scientific) using a gradient of 2% to 60% mobile phase B (80% ACN) for 20 min, increased to 95% mobile phase B over 10 min, and maintained at 95% mobile phase B for 15 min, and electro-sprayed at 300 nL/min into the Q-Exactive HF. Data-dependent spectra were acquired automatically (automated switching ESI mode) via high-resolution (60,000) precursor ion scan over a full mass scan range of *m*/*z* 67−1000. The source ionization parameters were optimized for a transfer temperature at 300 °C and a spray voltage set to 2.1 kV and −1.8 kV for the positive and negative modes, respectively. MS2 scans were performed at 15,000 resolution, with a maximum injection time of 64 ms, using stepped normalized collision energies (NCEs) of 10, 20, and 40. Dynamic exclusion was enabled using a time window of 10s. Raw data (switching mode) were split into positive and negative files and subject to OmicsNotebook (R script) for peak detection, deconvolution, retention time alignment, and metabolite identification against open database [25]. Intensities of features (putative metabolites) were normalized prior to differential analysis (moderated Student *t*-test).

Live-Cell Calcium Imaging—On day 30+ of differentiation, WT and mutant hiPSC-CMs were plated at 80k cells/well in a 24-well plate. Three days after plating the hiPSC-CMs, they were washed three times in Tyrode’s buffer (Thermo Fisher Scientific) and incubated in 10 μM Rhod-3 AM calcium indicator, PowerLoad, and Probenecid for 45 min covered from light, following the protocol from the Rhod-3 AM calcium imaging kit (ThermoFisher). Cells were washed in Tyrode’s buffer and incubated in Probenecid for an additional 45 min. Cells were then washed in Tyrode’s buffer three additional times and kept in Tyrode’s during live imaging. Calcium transients were acquired at 30 frames per second at 6X on a Nikon Eclipse Ti (Nikon Instruments, Tokyo, JP) with an Evolve EMCCD Camera (Photometrics, Tucson, AZ, USA), equipped with a temperature and CO2 equilibrated environmental chamber. hiPSC-CMs were electrically stimulated at 1 Hz using a C-Pace EP stimulator (IonOptix, Westwood, MA, USA). Videos were acquired at 15 locations per experimental group with a 560 nm laser illumination wavelength. Calcium transients of each cell were calculated using a custom Matlab script tracking intensity change over time within the cell. Calcium release was calculated as the time it took the calcium intensity in the cytoplasm to reach 50% maximum intensity, while calcium reuptake was calculated as the time it took the calcium intensity in the cytoplasm to decrease to 50% of its maximum intensity. This was repeated over three differentiations (*N* = 3), with n > 23 cells per differentiation. SR storage was determined from the ratio of the calcium transient immediately following and preceding incubation with the addition of 20 mM caffeine to Tryrode’s buffer (final concentration of 10 mM). All data were normalized to the average WT values for the matched differentiation. Data were assessed with a two-way ANOVA with a mixed-effect model that was corrected for multiple comparisons using a Tukey test with a 95% confidence level.

Targeted C13 Flux and BPTES application—C13 metabolomics flux metabolomics analyses were performed according to Yuan and collaborators [117]. Briefly, hiPSCs-derived cardiomyocytes, at day 30, were cultured during 24 h in RPMI glucose free + B27+ and 13C6 glucose (CLM-1396) added at a final concentration of 11.1 mM for glucose flux or cultured in RPMI glutamine free + B27+ and 13C5 glutamine (CLM-1822) added at a final concentration of 2.05 mM for glutamine flux. Metabolites were extracted with −80 °C cold 80% methanol (LC-MS grade), centrifuged, and supernatant was dried on speedvac over 18 h. Metabolite pellets were stored at −80 °C up to analysis (no more than 7 days) and protein pellets were used to normalize metabolites levels. Targeted metabolomics was performed using SRM LC-MS (^13^C glucose: *N* = 5 and ^13^C glutamate: *N* = 8, where *N* = number of differentiations). In cases of no analyte measurement (i.e., below detection limit) in a given sample, analyte values were excluded for that sample. All possible 13C labeled transitions were monitored. BPTES inhibition of glutamine oxidation was performed according to the manufacturer’s instructions (Agilent Seahorse XF Mito Fuel Flex Test Kit), 2 differentiations. Contractile parameters were assessed according to Toepfer and collaborators [118]. Data were assessed via unpaired, two-tailed *t*-test with Welch’s correction. The schematic in panels (B) and (C) was created in reference to Figure 1 of Fan et al. and Mcdonald et al. [119,120].

## Figures and Tables

**Table 1:** Patient information.

Sample ID	Age	Sex	MYH7 Mutation
Control 1	23	F	-
Control 2	45	F	-
Control 3	57	M	-
Control 4	43	M	-
Control 5	67	F	-
HCM 1	37	F	Met849Thr
HCM 2	51	F	Asp906Gly
HCM 3	46	M	Val606Met
HCM 4	29	M	Arg143Gln
HCM 5	65	F	Gly584Arg

## Data Availability

Project accession: PXD039613. Project doi: 10.6019/PXD039613.

## Supplementary Materials

The following supporting information can be downloaded at: https://www.mdpi.com/article/10.3390/ijms24054724/s1.
