# Supplementary material for: Multi-Omics Profiling of Hypertrophic Cardiomyopathy Reveals Altered Mechanisms in Mitochondrial Dynamics and Excitation–Contraction Coupling
Source: Int J Mol Sci. 2023 Mar 1;24(5):4724. doi: 10.3390/ijms24054724 (PMC10002553; doi:10.3390/ijms24054724)
Supplement: Supplementary file 1 [file ijms-24-04724-s001.zip › ijms-2205022-Figures S1 and S2.pdf]

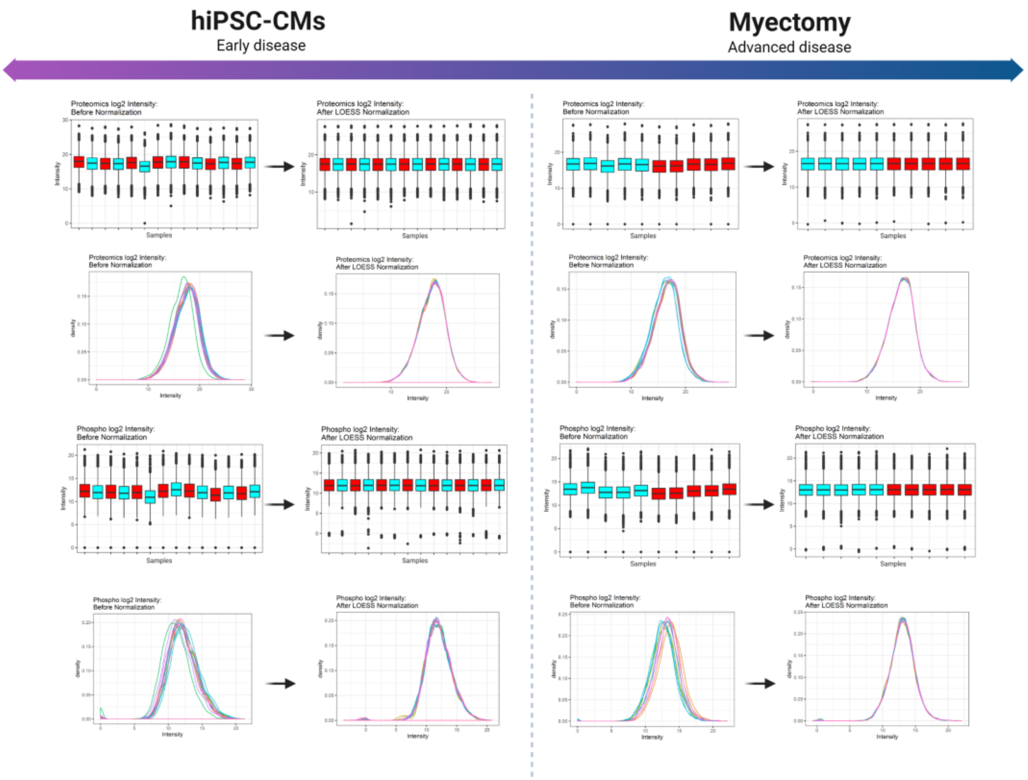

Figure S1. Intensity plots of proteome and phosphoproteome for hiPSC-CM and myectomy specimens.

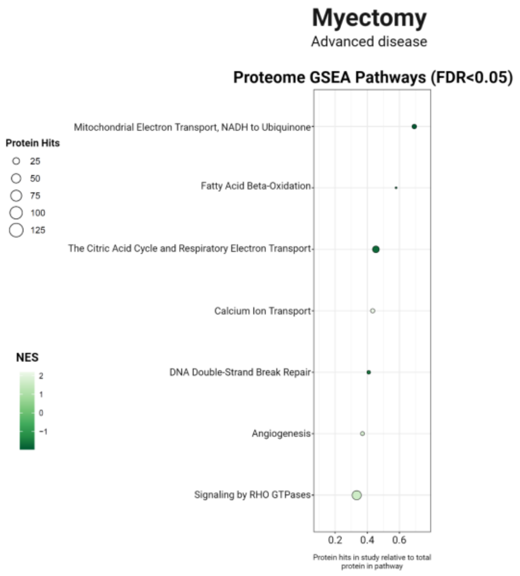

Figure S2. Top differential pathways from gene set enrichment analysis of mutant versus control myectomy proteome.
