# Supplementary material for: Multi-Omics Profiling of Hypertrophic Cardiomyopathy Reveals Altered Mechanisms in Mitochondrial Dynamics and Excitation–Contraction Coupling
Source: Int J Mol Sci. 2023 Mar 1;24(5):4724. doi: 10.3390/ijms24054724 (PMC10002553; doi:10.3390/ijms24054724)
Supplement: Supplementary file 1 [file ijms-24-04724-s001.zip › ijms-2205022-supplementary captions.pdf]

Supplementary Table S1: hiPSC-CM expression values. (A) Proteomics and (B) Phosphoproteomics data. The exact phosphosite is provided in (B) amino acid column. Blue samples are R403Q mutants and red are WT.

Supplementary Table S2: hiPSC-CM gene set enrichment analysis (GSEA) pathways. A positive NES denotes enrichment in HCM.

Supplementary Table S3: Patient myectomy expression values. (A) Proteomics and (B) Phosphoproteomics data. The exact phosphosite is provided in (B) amino acid column. Red samples are MYH7 mutants and Blue are WT.

Supplementary Table S4: Patient myectomy gene set enrichment analysis (GSEA) pathways. A positive NES denotes enrichment in HCM.

Supplementary Table S5: hiPSC-CM metabolic enrichment network analysis (MOMENTA) pathways. A positive NES denotes enrichment in HCM.

Supplementary Table S6: hiPSC-CM global metabolomics.

Supplementary Table S7: hiPSC-CM ^{13}C labeled glucose measurements.

Supplementary Table S8: hiPSC-CM ^{13}C labeled glutamine measurements.

Supplementary Table S9: Patient myectomy metabolic enrichment network analysis (MOMENTA) pathways. A positive NES denotes enrichment in HCM.

Supplementary Table S10: hiPSC-CM and patient myectomy excitation contraction coupling phosphoprotein expression values for figure 5.

Supplementary Figure S1: Intensity plots of proteome and phosphoproteome for hiPSC-CM and myectomy specimens.

Supplementary Figure S2: Top differential pathways from gene set enrichment analysis of mutant versus control myectomy proteome.
